# Gestational Hypertriglyceridemia Management and the Utility of Multidisciplinary Care

**DOI:** 10.1016/j.jaccas.2025.106522

**Published:** 2026-01-14

**Authors:** Brooke Zlotshewer, Jared A. Spitz, Lily N. Dastmalchi

**Affiliations:** a Lewis Katz School of Medicine at Temple University, Temple University Hospital, Philadelphia, Pennsylvania, USA; b Inova Schar Heart and Vascular, Fairfax County, Virginia, USA

**Keywords:** cardio-obstetrics, dyslipidemia, gestational hypertriglyceridemia, hypertriglyceridemia, lipidology

## Abstract

**Background:**

Severe hypertriglyceridemia (HTG) in pregnancy can lead to adverse outcomes such as preeclampsia, preterm delivery, and pancreatitis. However, there are no official management guidelines for gestational HTG.

**Case Summary:**

A 33-year-old woman at 32 weeks of gestation presented to the lipid clinic for severe HTG. With a multidisciplinary care team, a treatment plan including fenofibrates and icosapent ethyl was initiated. The treatment plan successfully reduced triglycerides and led to an uncomplicated delivery.

**Discussion:**

Gestational HTG can pose severe risks to pregnant patients. Limited safety data exist on treatment options in pregnant patients; thus there are no official management guidelines for treating this condition. Future work is needed to establish official guidelines for gestational HTG and preconception counseling to protect the mother and fetus.

**Take-Home Message:**

Given the treatment barriers of HTG in this vulnerable population, we present a case where multidisciplinary care successfully managed severe HTG in pregnancy.

## Graphical Abstract

**Figure:**
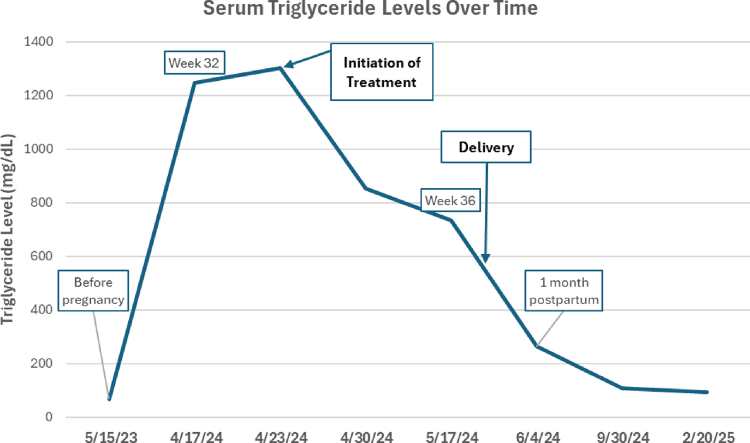

## History of Presentation

A 33-year-old G1P0 woman at 32 weeks of gestation was referred to the lipid clinic for evaluation of severe hypertriglyceridemia (HTG) during pregnancy.

**Take-Home Messages**

• This case demonstrates the importance of multidisciplinary care in successfully treating gestational HTG.
• Official management guidelines are necessary for gestational HTG to reduce morbidity and mortality in this vulnerable and at-risk patient population.

She had normal hemoglobin A1c, fasting glucose, and glucose tolerance testing results.

## Past Medical History

She had a history of untreated dyslipidemia (DLD), in which her cholesterol and low-density lipoprotein cholesterol (LDL-C) had been persistently elevated for 7 years before pregnancy. One year before pregnancy, the patient had a lipid panel with an elevated cholesterol level of 252 mg/dL, an elevated LDL-C level of 171 mg/dL, a high-density lipoprotein cholesterol (HDL-C) level of 71 mg/dL, and a normal triglyceride (TG) level of 68 mg/dL. She was never on therapy before, nor was therapy discussed previously, given her age. She did not receive preconception counseling pertaining to her history of DLD.

Her family history is notable for DLD in both parents and myocardial infarction in her father during “middle age,” though the exact age was not recalled. The patient was uncertain regarding her grandparents' lipid profiles.

## Investigations

Her initial routine prenatal lipid panel was ordered by her primary care physician at 32 weeks of gestation, showing TG 1,247 mg/dL, total cholesterol 329 mg/dL, HDL-C 56 mg/dL. LDL-C could not be calculated given the elevated HTG.

## Management

She was referred to a maternal fetal medicine physician, who consulted the cardio-obstetrics group and the advanced lipid clinic. The teams discussed the case and agreed on a treatment plan. Icosapent ethyl (2 g twice a day) and a fenofibrate (145 mg daily) were prescribed after discussion of risk and benefit. She was counseled by her lipidologist to begin a low-fat and low-carbohydrate diet with referral to a nutritionist for further guidance.

## Outcome and Follow-Up

**Figure 1:**
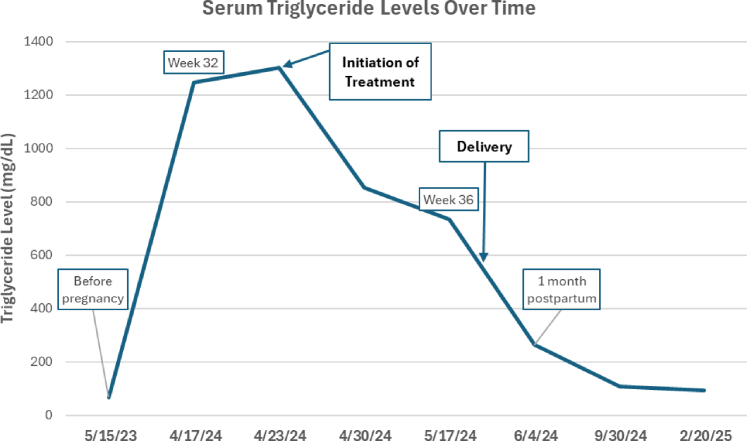
Serum Triglyceride Levels Over Time in Treatment of Gestational Hypertriglyceridemia

After intensive treatment initiation, her TG decreased to 733 mg/dL at 36 weeks of gestation (Figure 1). She had an uncomplicated delivery at 37 weeks. Her TG-lowering medications were held before delivery and not restarted postpartum to allow for breastfeeding.

One month postpartum, her TG decreased to 264 mg/dL, and she no longer required TG therapy. At 4 months postpartum, her LDL-C was 229, lipoprotein(a) was 162, TG was 94, and non–HDL-C was 79 mg/dL. Genetic testing with a familial hypercholesterolemia panel was negative. She was started on rosuvastatin 10 mg, with an improvement in LDL-C to 76 mg/dL. The patient has been contemplating a future pregnancy, and thus, there will be ongoing preconception counseling.

Preconception counseling will include discontinuation of her statin medication 4 weeks before pregnancy attempt. Throughout pregnancy, serial lipid panels will be followed, especially in the second trimester, which may trigger restarting medications in the mid-late second trimester and beyond. Additional counseling will be provided on dietary changes during pregnancy to minimize saturated fat and carbohydrates to mitigate any rise in TGs while still being mindful of caloric needs during pregnancy. These diet changes will also aid in risk reduction of preeclampsia, which can be associated with elevated TGs during pregnancy.

## Discussion

DLD in pregnancy poses many health risks, such as gestational diabetes, cardiometabolic dysfunction in the fetus, and increased cardiovascular disease. Professional society guidelines for the management of this condition do not exist. We present a case that demonstrates an effective strategy to manage HTG in pregnant patients, for which guidelines do not exist.

Untreated HTG, especially TG above 250 mg/dL, can lead to pancreatitis and preeclampsia, compromising placental blood flow to the fetus.^1^ TG increases 2- to 3-fold by the third trimester.^2^ The increase in TG during pregnancy is a physiologic metabolic adaptation that allows growth and development of the fetus. During the second trimester of pregnancy, the mother enters a catabolic state, which increases insulin resistance, leading to increased availability of fatty acids and therefore an increase in plasma TGs. Although this is a physiologic change, there can be severe adverse outcomes if the TG level becomes too elevated.^3^ Although those diagnosed with severe HTG in pregnancy can have preexisting HTG, many present for the first time during pregnancy.^2^ Thus, HTG should be addressed to mitigate morbidity and mortality for the mother and fetus.^4^

With there being no societal guidelines on management of these conditions, the National Lipid Association and American Heart Association have published statements addressing this crucial topic, stating that very high TG levels (>500 mg/dL) increase the risk of pancreatitis and may benefit from pharmacological agents during the second trimester, second trimester being after embryogenesis occurs.^2^^,^^5^ Without guidelines, practitioners must gather data from sources to treat the mother and protect the fetus. Pregnant women are rarely included in clinical trials given the risk of teratogenicity. Although studies show that fenofibrates and niacin are safe in pregnancy, limited safety data exist. In 2021, the Food and Drug Administration asked for black box warning removal to allow statin use in pregnancy for special conditions, such as familial hypercholesterolemia.^6^ To ensure early identification, we should screen those with a history of pancreatitis, abdominal pain with prior estrogen use, and family history of HTG.^2^

**Figure 2:**
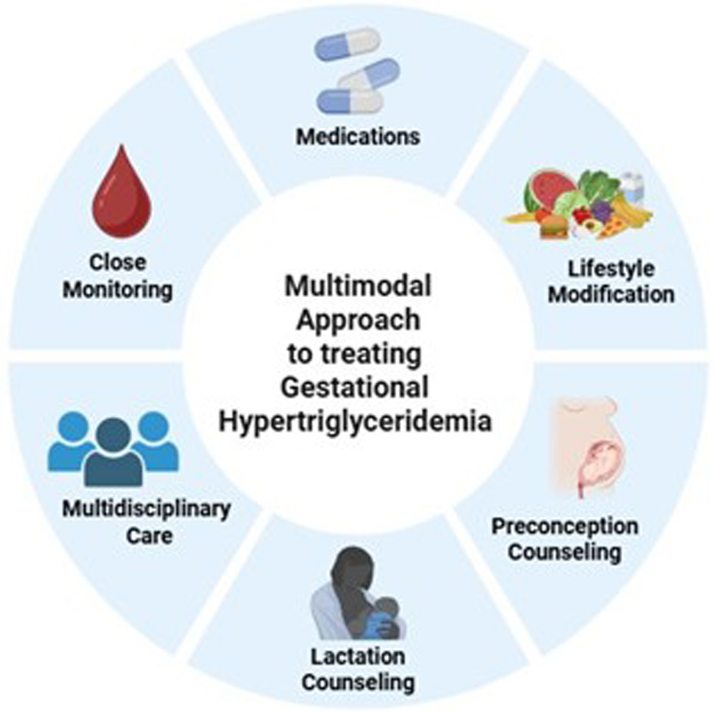
Multimodal Approach for Treating Gestational Hypertriglyceridemia

To optimally treat gestational HTG, a multimodal approach is required (Figure 2), including lifestyle modifications, medications, and close monitoring. Treatment should incorporate multidisciplinary care with collaboration between cardiology, maternal-fetal medicine, lipidology, and nutrition, which is essential to optimize both maternal and fetal outcomes. In patients interested in pregnancy who have a history of DLD, there should be efforts to optimize modifiable factors, such as nutrition and physical activity, and improve lipid levels before pregnancy. A plan should be in place to closely monitor lipids, with referrals made to specialists for close follow-up and multidisciplinary care. In addition, there should be a discussion of possible benefits or risks of discontinuation or initiation of lipid-lowering medications.^4^^,^^6^ In the postpartum period, lactation counseling is also an essential component of care for optimizing medications that are safe during lactation. We have included a summary of lipid-lowering medications and the current recommendations for their use during pregnancy and lactation (Table 1).

**Table 1:** Lipid-Lowering Medications in Pregnancy and Lactation^2^^,^^7^

Medication Class	Pregnancy	Lactation
Statins	Generally, should be stopped, except in select high-risk (HeFH and HoFH) cases. If the decision is made for statin therapy, it should be started during the second trimester and hydrophilic pravastatin is considered	Contraindicated
Bile acid sequestrants	Considered safe to use if TG <400 mg/dL	Considered safe to use if TG <400, but may benefit from vitamin supplementation
Fibrates	Considered during the second trimester if TG ≥500 mg/dL or if there is a history of pancreatitis associated with TG ≥1,000 mg/dL	Can resume breastfeeding 5 days after the final dose
Ezetimibe	Should be avoided	Should be avoided
Bempedoic acid	Should be avoided	Should be avoided
Omega-3 fatty acids	Considered during the second trimester if TG ≥500 mg/dL or if there is a history of pancreatitis associated with TG ≥1,000 mg/dL (added to omega-3 pregnancy supplements)	Considered if TG ≥500 mg/dL or if there is a history of pancreatitis associated with TG ≥1,000 mg/dL
PCSK9 inhibitors	Should be avoided	Should be avoided

HeFH = heterozygous familial hypercholesterolemia; HoFH = homozygous familial hypercholesterolemia; PCSK9 = proprotein convertase subtilisin/kexin type 9; TG = triglycerides.

This case shows how diet modification, icosapent ethyl, and fenofibrates in combination with close monitoring and multidisciplinary care can be an effective regimen to successfully lower TG, ensuring a positive outcome in a vulnerable patient. Future work is needed to measure medication safety profiles and for the development of guidelines for severe HTG in pregnancy.

## Conclusions

Severe HTG in pregnancy can have severe complications for the mother and fetus; thus early intervention is critical. Without formal guidelines, management of gestational HTG remains based on expert consensus and case reports, underscoring the need for individualized care. Multidisciplinary care allows for multimodal treatment. Future studies are needed to clarify medication safety and guidelines for severe HTG in pregnancy. American College of Cardiology/American Heart Association guidelines are necessary for gestational HTG to reduce morbidity and mortality in this vulnerable and at-risk patient population.

## Funding Support and Author Disclosures

Publication of this article was funded in part by the Temple University Libraries Open Access Publishing Fund. Dr Dastmalchi served on the Novartis advisory board in November 2023. All other authors have reported that they have no relationships relevant to the contents of this paper to disclose.
